# Bronchiolitis: evidence-based management in high-risk infants in the intensive care setting

**DOI:** 10.1038/s41390-024-03340-y

**Published:** 2024-06-20

**Authors:** Ruth Walsh, Liam Costello, Alexandria DiCosimo, Anne-Marie Doyle, Laura Kehoe, Cormac Mulhall, Sean O’Hara, Basil Elnazir, Judith Meehan, Eman Isweisi, Gergana Semova, Aoife Branagan, Edna Roche, Eleanor Molloy

**Affiliations:** 1 Discipline of Paediatrics, School of Medicine, Trinity College Dublin, the University of Dublin, College Green, Dublin, 2 Ireland; 2 Respiratory Medicine, Children’s Health Ireland at Tallaght, Tallaght University Hospital, Dublin, 24 Ireland; 3 Trinity Research in Childhood Centre (TRiCC), Trinity College Dublin, Dublin, Ireland; 4 Paediatrics, The Coombe Hospital, Dublin, 8 Ireland; 5 Endocrinology, Children’s Health Ireland at Tallaght, Tallaght University Hospital, Dublin, 24 Ireland; 6 Neurodisability Children’s Health Ireland at Tallaght, Tallaght University Hospital, Dublin, 24 Ireland; 7 Neonatology, Children’s Health Ireland at Crumlin, Dublin, 12 Ireland

## Abstract

**Aim:**

Systematically review the management of infants with severe bronchiolitis in a paediatric intensive care unit (PICU) setting with a focus on high-risk infants to identify gaps in evidence-based knowledge.

**Methods:**

This systematic review utilised Preferred Reporting Items for Systematic Review and Meta-analysis Protocols (PRISMA-P) to examine the literature on the PICU management of bronchiolitis in infants <24 months old. Three databases, Embase, PubMed and Medline, were searched and higher levels of evidence I, II and III were included.

**Results:**

There were 455 papers reviewed and 26 met the inclusion criteria. Furthermore, 19 of these studied respiratory interventions such as positive airway pressure and oxygen delivery. The remaining 7 examined: erythropoietin, caffeine, dexamethasone, protein supplementation, ribavirin, respiratory syncytial virus immune globulin, or diuretic therapy. Of the 26 studies, 20 excluded infants with high-risk conditions. Therapies showing favourable outcomes included Heliox, prophylactic dexamethasone pre-extubation, protein supplementation, and diuretic use.

**Conclusions:**

Clinical trials for bronchiolitis management frequently exclude high-risk children. Innovative study design in the future may improve access to clinical trials for the management of bronchiolitis in high-risk infants in a PICU setting.

**Impact:**

- Clinical trials for bronchiolitis management frequently exclude high-risk children.
- We review the evidence base for the management of an under-investigated patient demographic in the setting of acute bronchiolitis.
- Randomised controlled trials are needed to determine the efficacy of management strategies for bronchiolitis in high-risk infants in a paediatric intensive care setting.

## Introduction

Bronchiolitis is an acute viral inflammation of the bronchioles and is a leading cause of hospitalisations in young children.^1^ Respiratory syncytial virus (RSV) is the most common causative agent of infection, accounting for 50–80% of bronchiolitis cases.^2^ Approximately 2–3% of all children younger than 12 months of age are hospitalised with a diagnosis of bronchiolitis.^3^ Bronchiolitis is associated with substantial morbidity in both inpatient and outpatient settings.^4^ Most children hospitalised with acute bronchiolitis do not require further escalation of treatment, however, approximately 2–6% require admission to a paediatric intensive care unit (PICU), with 2-3% requiring invasive mechanical ventilation.^5^

A variety of factors are associated with the risk of more severe illness and increased risk of hospitalisation. Younger chronological age is the single most important predictor of severe bronchiolitis.^6^ Approximately two-thirds of RSV infant hospitalisations occur within the first five months of life.^4,6^ Additionally, prematurity (<36 weeks’ gestation), increases the risk of hospitalisation.^4,7^ The risk of severe bronchiolitis is higher among premature infants <29 weeks of gestation than those born at 29 weeks of gestation or later.^4^ Furthermore, this increase in risk is associated with the diagnosis of bronchopulmonary dysplasia (BPD) at 36 weeks (corrected gestational age), regardless of the gestational age at birth.^8^ Other high-risk medical conditions that increase the risk of severe bronchiolitis include previous episodes of wheeze such as previous bronchiolitis, congenital abnormalities such as Down Syndrome, chronic pulmonary disease such as BPD, cardiac disease such as a congenital heart defects, chronic renal disease, diseases of immunodeficiency, cancer, and sickle cell anaemia.^4,7,9^ These high-risk infants have an approximately five times greater rate of hospitalisation when compared to infants with no high-risk disease.^10^ Additionally, the presence of a pre-existing high-risk disease is associated with increased risk of death from severe infection.^11^

Randomised controlled trials (RCTs) have been conducted to determine the effectiveness of various agents in the management of this condition.^12^ These include the use of corticosteroids, inhaled beta-agonists, epinephrine, hypertonic saline, in addition to others, however, at present there is no single definitive treatment available for bronchiolitis.^12^ In turn, various guidelines have been developed for the management of bronchiolitis in low-risk patients.^13–16^ These serve as a guide for management; however, recommended treatments remain essentially supportive with emphasis placed on ensuring adequate hydration and oxygen supplementation as needed.^3,13^ Despite evidence-based guidelines for the treatment of bronchiolitis, wide variations in practice remain, including marked variability in the inpatient management of bronchiolitis across different sites.^17^

The aim of the present study was to systematically identify and characterise the current body of evidence available on the management options for patients with severe bronchiolitis who required escalation of treatment to a PICU setting. The systematic search was conducted to identify any potential gaps in the current literature pertaining to the management of this patient population.

## Methods

### Protocol

A protocol for this systematic review was generated to delineate the rationale, hypothesis, objectives, and methodology of the review (Supplemental Fig. 2). The Preferred Reporting Items for Systematic Review and Meta-analysis Protocols (PRISMA-P) guidelines were utilised to form the protocol.^18^ No Institutional Research Board (IRB) approval or consent was required for this study as it is a systematic review of previously published literature.

### Eligibility criteria

Studies were utilised for the review if they met all of the following inclusion criteria: Involved a study population of infants under 24 months of age; The population must have a clinical diagnosis of bronchiolitis; The population must be undergoing intensive care management.

Studies were excluded from this review if they met any of the following exclusion criteria: Not available in the English language; Did not have full texts available for full-text review; Did not report outcomes of the management used; The study did not take the format of a clinical study, such as a randomised controlled trial, cohort study or case-control study.

### Information sources and search strategy

In order to assess the evidence base for the management of these infants, a systematic review of the evidence for the intensive care management of bronchiolitis was conducted. Due to the wide variety of conditions which infer a higher risk of severe bronchiolitis, it was decided that independent searches for each condition would be unsuitable. Thus, we conducted our search for infants who required escalation to intensive care as those who require this level of intervention are typically those with a high-risk condition. For this review, articles were sourced from three online database search engines: PubMed, Medline, and EMBASE. The search strategy combined key terms and Emtree to source studies that incorporated the key aspects of our question; bronchiolitis and intensive care management. There was not a timeframe included in the search strategy as many older studies may still guide clinical management today. The last search was performed on the third of December 2021. The search strategy utilised is shown in Supplementary Fig. 1.

### Study selection

**Fig. 1:**
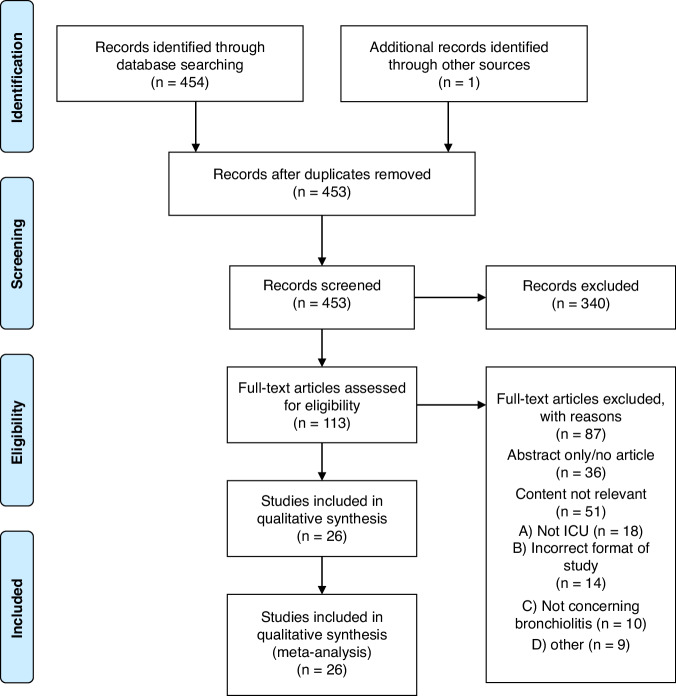
PRISMA flow diagram of study selection process. ICU Intensive Care Unit.

Following the search of the relevant databases for studies regarding the intensive care unit (ICU) management of bronchiolitis, all studies found were exported to EndNote 20 where duplicates were excluded.^19^ All studies were then exported to Covidence for screening by reviewers^20^. Studies were randomly divided between pairs of reviewers (R.W. and L.C, C.M. and L.K., AM.D. and S.O’H).^20^ The title and abstract of each study were screened independently by the allocated group in accordance with established eligibility criteria. Each study was reviewed by two of the reviewers to determine eligibility. Following this, full texts were independently screened in accordance with established eligibility criteria. At this stage, there was a search within each paper being reviewed for potential other relevant literature, these are included within the section on additional records identified through other sources (Fig. 1). Arbitration of any disputes regarding the inclusion or exclusion of papers was held with a team member uninvolved in the initial screening (A.D.) who was responsible for making the final decision.

### Data extraction

To extract the relevant data from the studies selected, they were divided amongst the reviewers to independently analyse their full texts to ascertain the details of the study including the type and population of the study, the intervention being assessed, the infants they excluded, and the results and outcomes of the study. The data was then reviewed blindly by one of the reviewers (R.W.). Any disputes which remained following this process were arbitrated by another author (S.O’H.) to give a final decision. The details from each paper such as the study type, population, intervention, exclusion criteria, results, and outcomes were exported from Covidence to Excel for comparative and statistical analysis.^20^ Due to significant heterogeneity across the limited number of included studies, a meta-analysis was not considered appropriate and instead a qualitative summary of findings was presented in a table of results (Supplementary Table 2).

### Risk of bias

The revised Cochrane risk-of-bias tool for randomised trials and the Newcastle-Ottawa Scale were used by reviewers to screen for the risk of bias within the selected studies and critically appraise these studies.^21,22^ These tools search for bias across distinct domains, with conclusions from each domain revealing the study’s risk of bias. Often, in the case of incomplete reporting, conclusions could not be assessed, thus these domains were reported as no information. The outcome of this risk of bias tool is summarised into Supplementary Table 3.

## Results

Following review, of the 455 papers retrieved in our systematic search for clinical studies pertaining to the ICU management of severe bronchiolitis, 26 papers were identified which were deemed relevant to the aim of this review. The study selection process undertaken can be seen in the preferred reporting items for systematic review and meta-analysis (PRISMA) flow chart diagram in Fig. 1.^18^ The search criteria in Supplementary Fig. 1 produced 454 studies that were exported to Covidence from the PubMed, MedLine and EMBASE databases.^20^ One further study was identified as a citation from a reviewed text. After the removal of duplicate studies (*n* = 2), title and abstract screening was undertaken on the remaining 453 studies. 340 of these were removed as they met the established exclusion criteria outlined in the protocol above. Full-text screening was performed on the remaining 113 studies. 87 of these were excluded for reasons including not meeting selection criteria, incorrect study design, or incorrect patient cohort (not bronchiolitis patients treated in intensive care). The management interventions identified and included in this review, in addition to the number of studies were included on each intervention (Supplementary Table 1).

Fifteen of the 26 studies were RCTs and 10 were cohort studies, one study was a prospective interventional trial. All of these assessed infants with bronchiolitis undergoing intensive care management. Nineteen of the 26 studies examined respiratory interventions such as positive airway pressure and oxygen delivery supports such as high-flow nasal cannula (HFNC) and CPAP. Seven of the 26 studies examined multiple other methods of management including erythropoietin (EPO), caffeine, dexamethasone, protein and energy intake, ribavirin, RSV immune globulin as well as the use of diuretics.

Of the 26 studies we examined, 20 excluded patients with pre-existing medical conditions, such as underlying respiratory and cardiovascular disease, similar to the trials of the National Institute for Health and Care Excellence (NICE) guidelines (2015) (Table 1).^14^ Risk of bias was assessed according to the protocol (Supplementary Table 3). The details for each of the domains assessed in both the cohort studies and RCTs can be found in Supplementary Table 2. The risk of bias would be considered low-moderate, so overall the evidence from the studies included in this review would be moderate-high grade evidence.

**Table 1:** Summary of the excluded medical conditions within the Randomised Controlled Trials.

Exclusion criteria	Chest physiotherapy (7)	Antibiotics (7)	Hypertonic saline (18)	Inhaled bronchodilators (20^a^)	Systemic corticosteroids (4)	Combined bronchodilator and corticosteroid therapy (8)	Montelukast (2)	Heliox (5^b^)	Oxygen supplementation (3)	Fluid and nutritional support (2)	Additional recent studies^c^ (9)	Percentage excluded^d^
**Critically Ill (Unspecific)**	0	0	2	5	3	3	0	1	3	0	4	**22.35%**
**Prematurity**	2	1	9	10	2	2	0	0	0	0	2	**32.94%**
**Low birth weight (<2500** **g)**	0	0	1	1	0	0	0	0	0	0	0	**2.35%**
**Respiratory**	5	4	17	19	3	8	2	4	2	2	9	**88.24%**
**Cardiovascular**	5	5	17	18	3	6	2	1	2	1	7	**78.82%**
**Neurological**	4	1	2	5	0	2	0	0	2	1	5	**25.88%**
**Gastrointestinal**	1	0	1	3	3	0	0	0	0	0	1	**10.59%**
**Haematological**	1	0	0	1	2	0	0	1	0	0	1	**7.06%**
**Renal**	0	0	2	1	0	0	0	0	0	0	2	**5.88%**
**Hepatology**	0	0	0	1	3	0	0	0	0	0	2	**7.06%**
**Immunodeficiency**	1	3	7	8	3	3	1	0	0	0	3	**34.12%**
**Metabolic disease**	0	0	1	1	0	0	0	0	0	0	2	**4.71%**
**Congenital anomaly (eg. Down syndrome)**	1	0	1	0	0	0	0	0	1	0	3	**7.06%**
**Radiographic findings of pulmonary disease (eg. consolidation, atelectasis, etc.)**	1	0	1	5	0	2	0	0	1	0	2	**14.12%**
**Prior medication or treatment (bronchodilators, corticosteroids, etc.)**	1	3	11	15	4	6	1	3	0	0	1	**52.94%**
**Escalation of and/or PICU**^**e**^ **Management (eg. requiring mechanical ventilation, resuscitation)**	4	6	15	8	1	1	1	2	1	2	2	**50.59%**
**Atopy (wheezing, atopic dermatitis, allergic rhinitis, asthma, etc.)**	2	3	17	8	4	6	0	1	0	0	0	**48.24%**
**Associated Infection**	0	2	1	4	2	2	0	0	0	0	0	**12.94%**
**History of the previous episode**	2	3	1	9	4	4	1	1	1	0	0	**30.59%**
**Poor vital signs or stats (oxygen saturation, CS score, obtunded consciousness)**	0	1	10	7	0	2	1	1	1	2	0	**29.41%**
**Inability to receive required interventions (eg. nebulised medications)**	1	1	2	1	2	2	0	1	0	0	0	**11.76%**
**Other (eg. informed consent, language barrier, age)**	3	2	6	3	3	4	0	1	0	1	3	**30.59%**

Bold values: The right hand column entitled “percentage excluded” is the percentage of all trials in the NICE guidelines that excluded the given condition in that row.

^a^Inhaled Bronchodilator papers totaled 24; 4 full-text reviews were unavailable.

^b^Heliox papers totaled 6; 1 full-text review was unavailable.

^c^Additional studies were found doing a search of relevant randomised control trials (RCTs) conducted since the publication of the NICE guidelines in 2015 (National Institute for Health and Care Excellence, 2015).

^d^Calculated by determining the number of studies that excluded a specific group of patients, divided by the total number of studies included in the NICE Guidelines 2015

^e^PICU: Paediatric Intensive Care Unit.

On review of the RCTs included in the NICE guidelines (2015), the majority of these trials excluded those infants with any high-risk condition, comorbidity, or requirement for escalation of care (Table 1).^14^ The greatest percentage of patients excluded from the clinical trial data included those with underlying respiratory disease (88.24%), followed by those with underlying cardiovascular disease (78.82%). Additionally, a large percentage of patients were excluded for having received previous medication or treatment (52.94%), such as bronchodilators or corticosteroids, as well as having any history of atopy including asthma, wheeze, atopic dermatitis, or allergic rhinitis (48.24%). Approximately half (50.59%) of the studies excluded patients who required escalation of care and/or PICU management. Finally, over 30% of studies excluded those who were premature and around 7% of studies excluded congenital anomalies such as Down Syndrome.

## Discussion

Research on the care of critically ill high-risk infants with bronchiolitis has been limited to date.^23^ Despite the existence of national guidelines pertaining to the management of acute bronchiolitis, little impact has been shown for the management of patients in the PICU with severe disease.^24^ For example, the NICE guidelines recommended against the use of nebulised therapies for the management of patients in the PICU and advised the use of continuous positive airway pressure (CPAP) in patients with impending respiratory failure.^14^ However, small regional studies published since the 2015 NICE guidelines have found that the use of nebulised therapies, including hypertonic saline, have increased.^24^ Additionally, non-invasive ventilation (NIV) such as high-flow oxygen therapy has increased, while the use of CPAP decreased.^24^

Available guidelines rarely included specific guidance for the management of infants with bronchiolitis in the PICU setting.^1^ Significantly, acute bronchiolitis accounts for around 13% of PICU admissions in the UK, contributing a significant burden on the availability of PICU beds.^1^ Additionally, it has been shown that those infants experiencing severe disease and admission to the PICU are those with identifiable risk factors including prematurity and chronic medical conditions as above.^1^ As shown in Table 1, the majority of RCTs included in the NICE guidelines (2015) excluded those infants with any high-risk condition, comorbidity, or requiring escalation of care.^14^ The greatest percentage of patients excluded from the clinical trial data included those with underlying respiratory disease, followed by those with underlying cardiovascular disease. Additionally, a large percentage of patients were excluded for having received previous medication or treatment, such as bronchodilators or corticosteroids, as well as having any history of atopy including asthma, wheeze, atopic dermatitis, or allergic rhinitis. Over half of the studies excluded patients who required escalation of care and/or PICU management. Finally, over 30% of studies excluded those who were premature and around 7% of studies excluded congenital anomalies such as Down Syndrome. Given the exclusion of high-risk infants from trials which are subsequently used to make evidence-based recommendations, this significantly limits the generalisability of the guidelines among infants with various underlying health conditions. Based on this observation, we hypothesize that there is a significant gap in the research pertaining to the management of high-risk infants experiencing bronchiolitis.

The National Institute for Health and Care Excellence (NICE) guidelines on bronchiolitis management and diagnosis in children were published in 2015 and serve as a cumulative management protocol developed from over 90 RCTs grouped based on treatment intervention.^14^ Using the findings of the RCTs, recommendations were made regarding the use of different management techniques. For example, regarding oxygen supplementation for babies and children with bronchiolitis, it is recommended that supplemental oxygen be administered to children aged 6 weeks and above, and to babies under 6 weeks of age or children of any age with underlying health conditions, if their measured oxygen saturations are persistently below 90% and 92%, respectively. However, the best method of oxygen delivery could not be determined. The recommendation of an oxygen saturation threshold of 90% in infants and children >6 weeks, for the provision of supplemental oxygen and discharge from the hospital, was largely based on the results of an RCT which compared an oxygen saturation threshold of 90% to a threshold of 94%.^25^ This RCT was conducted in patients >6 weeks of age with fewer comorbidities and less risk of poor outcomes compared to those children with bronchiolitis typically seen in a hospital setting.^25^ The NICE guideline notes that there was no evidence available on oxygen saturation targets for patients at higher risk of severe disease (patients aged <6 weeks and children of any age with underlying health conditions).^14^ Therefore, the decision to recommend a 92% threshold for high-risk patients was not evidence-based, but instead was made on professional judgement. Additionally, it is recommended to give fluids via nasogastric or orogastric tube when oral hydration is inadequate and escalate to isotonic intravenous fluids in those with impending respiratory failure. It states that chest physiotherapy should not be performed, however, consideration should be given when the child presents with comorbidities that lead to difficulties clearing secretions. It was also found that there was no clear benefit for the routine use of antibiotics. The available evidence on the use of hypertonic saline was also assessed, but it was found to not offer any reduction in hospital stay when compared to no treatment and therefore was not recommended. It was advised that upper airway suctioning should not be routinely performed for bronchiolitis in children but can be considered to alleviate distress when feeding difficulties are present. Additionally, in those presenting with apnoea, it is stated that upper airway suctioning is always recommended. Finally, two management techniques, Heliox - the gas mixture of helium and oxygen and Montelukast, were found to have limited and contradictory evidence and therefore further research was recommended.^26,27^

Infants with high-risk conditions have an increased likelihood of hospitalisation for bronchiolitis and are more likely to be encountered in a secondary or tertiary care setting compared to those with no underlying medical comorbidities.^10^ Despite the higher rate of hospitalisation and death, this search has confirmed that there is paucity of evidence pertaining to the PICU management of this patient cohort in the literature, echoing the findings of Lin and Madikans (2015).^23^

The vast majority of studies measured methods of oxygen delivery, which remains the mainstay of treatment for all patients with bronchiolitis with saturations below 92%.^14^ Although the need for supplemental oxygen is a universally accepted management, questions remain regarding the optimal method of delivery. When comparing HFNC and CPAP, Metge et al.^28^ found no difference between both modes of delivery in terms of the patient’s clinical parameters or length of PICU stay.^24^ Habra et al.^29^ conducted a larger study investigating NIV (CPAP and BiPAP), however, among the exclusion criteria were patients deemed high-risk with underlying respiratory or congenital heart disease^28^ Although a smaller study, the same exclusion criteria did not apply for the Metge et al.^28^ study, which only excluded infants with endotracheal intubation at time of admission to ICU.^24^ These findings are in contrast to those of Griffiths et al.^24^ which found a trend favouring high-flow oxygen therapy away from CPAP since the update of the NICE guidelines in 2015, however, this was studied in those with less severe disease prior to PICU admission.^14,24^ Despite these findings, the use of NIV was associated with favourable outcomes in several of the studies whereas HFNC was associated with greater patient tolerance and decreased risk of nasal injury.^30–35^

Heliox therapy utilises a helium-oxygen gas mixture and works by reducing airway resistance through decreasing turbulent flow and in turn reduces the work of breathing.^36^ This intervention also appears to have anti-inflammatory mechanisms.^37^ Heliox has been investigated for use in bronchiolitis management and was reported on in the NICE guidelines.^14^ However, they did not recommend its use due to limited and contradictory evidence and instead indicated the need for further research. Our search yielded two studies looking at the use of heliox therapy in the PICU management of bronchiolitis.^27,38^ In a small study conducted by Liet et al.^38^ heliox therapy did improve overall respiratory status in RSV bronchiolitis and decreased PICU length of stay.^38^ This reaffirmed the findings of Martinón-Torres et al.^26^ who had similar findings across a population of 38 critically ill infants.^27^ However, both these studies had relatively small sample sizes and research on this intervention for bronchiolitis has not been expanded. There may be limited recent research on this intervention as the use of high oxygen concentrations in those who are critically unwell with bronchiolitis is contraindicated, as well as the low-density gas causing issues in the ventilator readings.^39,40^

Management outside of ventilation strategies included a variety of more novel therapeutic methods. Those with positive results included the use of dexamethasone, nutritional supplementation, and diuretics. Dexamethasone administered prophylactically prior to extubation was shown to significantly decrease the risk of reintubation when compared to patients who did not receive a prophylactic dose.^41^ However, similar to the majority of studies reviewed, patients with upper airway pathology were excluded. Additionally, this study did not report on the possible adverse effects of dexamethasone, which includes hypertension, glucosuria, and immunosuppression. This may alter the risk-benefit ratio of administering this medication to high-risk patients who may be vulnerable to the side effects.

Nutritional supplementation, focusing on ensuring adequate protein anabolism and supplementation, was another management technique investigated in the PICU patient population. In this study by De Betue et al.^42^ 18 critically ill infants were given additional protein supplementation which ensured nutritional needs were met.^42^ They found that the patients who were given the protein-enriched formula had markedly higher protein balance and more favourable outcomes. In addition to this, the infants receiving the protein-enriched formula were significantly lower in gestational age than the control group, which along with the small population size, may threaten the credibility of the results. However, this may indicate a greater level of importance in adequate nutrition for critically ill infants with low weight at admission, especially as this is a well-established risk factor for poor outcomes.^43^

There is conflicting evidence on the use of furosemide in this clinical setting. A randomised controlled trial by Williamson et al.^44^ found no improvement in respiratory distress in infants with moderate to severe bronchiolitis treated with single-dose furosemide.^44^ However, in the study by Agasthya et al.^45^ furosemide was found to improve oxygenation in ventilated infants with severe bronchiolitis during fluid overload states.^45^ Furosemide reduced the need for prolonged mechanical ventilation, decreased PICU stay, and improved oxygenation. After 24 h, there were modest improvements in FiO_2_ with no evidence of hemodynamic instability or electrolyte disturbance. Future research in this area may produce an evidence base for this intervention during acute management.^46,47^ The potential decrease in the length of mechanical ventilation reduces the risk of other complications such as ventilator-associated infections, atelectasis, and pneumothoraces.^48^

Some interventions for the PICU management of bronchiolitis in high-risk infants did not display favourable outcomes. These included ribavirin, erythropoietin (EPO), caffeine, and RSV immune globulin (RSVIG). Aerosolised ribavirin showed a lack of effectiveness in treating RSV bronchiolitis across 41 patients.^49^ No differences were observed between the patients who received the ribavirin and the cohort who received the placebo (normal saline). In their 2003 paper, Jacobs et al. studied the clinical benefit of the administration of exogenous EPO for critically ill infants with bronchiolitis.^50^ EPO showed no difference in improvement when administered, quantified as the need for transfusions, between the two groups. However, the patients receiving EPO injections had marginally greater reticulocyte counts compared to the control patients. Heuzé et al.^50^ assessed the benefit of caffeine administration in infants with bronchiolitis-related apnoea in the PICU.^51^ However, in this study, the length of PICU stay and the number of patients requiring mechanical ventilation remained the same for both the caffeine and control groups. Lastly, an older study by Rodriguez et al.^51^ looked at whether RSVIG could be of benefit for the treatment of acute bronchiolitis.^52^ Although not significant, the administration of RSVIG resulted in fewer hospital days, but only in those infants with severe disease. Overall, these interventions were not of significant benefit in the management of bronchiolitis and additional large RCTs are needed to confirm these findings.

As is often quoted in medicine – Prevention is better than cure. In that respect, an imminent healthcare development that may drastically reduce the prevalence of severe bronchiolitis in high-risk infants is the implementation of RSV immunisation in children. The newest agent, Nirsevimab, is a respiratory syncytial virus F protein‑directed fusion inhibitor, which reduces the risk of severe RSV disease by approximately 80%.^53^ The implementation of this vaccine in many countries around the world is imminent as it is now recommended by the Centres for Disease Control and Prevention for all infants under 8 months of age, and high-risk infants aged 8-19 months.^53^ It protects a child for the duration of an RSV season (around 5 months).^52^ Palivizumab, a monoclonal antibody with similar mechanism of action but shorter duration of action than Nirsevimab, has been used for many years exclusively in the setting of high-risk infants under 24 months for the prevention of severe disease to great effect – reducing infection rates and associated hospitalisation^53^. The wider implementation of RSV vaccination in infants will hopefully reduce the morbidity and mortality associated with RSV infection in high-risk infants.

Overall, our study reported on papers with higher levels of evidence I, II and III, which strengthens the impact of the results. However, as was seen in the 2015 NICE guidelines; as well as other evidence-based guidelines include those from the American Association of Paediatrics, Canadian Paediatric Society, and The Royal Australasian College of Physicians; a large proportion of studies that guide management exclude patients on the basis of their medical comorbidities. A similar finding was seen among the studies identified in our search. This limits our results as, although studies on the management of patients in the PICU were identified, a significant proportion of those management techniques were not trialled on high-risk patients. Additionally, utilisation of our clearly defined search criteria may have limited our results such that only studies conducted on patients within the PICU setting were included, excluding high-risk patients that may have been managed on a ward or high dependency unit (HDU). Lastly, a large proportion of the studies reviewed had small sample sizes and therefore these management techniques should be examined in larger trials.

Finally, as previously mentioned, infants with high-risk conditions have an increased chance of being hospitalised for bronchiolitis.^10^ Despite this population experiencing more severe illness and morbidity, they have been excluded quite consistently from clinical studies. From the studies extracted for the purpose of this review, it is clear that a lack of evidence exists pertaining to the management of high-risk patients with bronchiolitis. It highlights the need for evidence-based trials with a particular preference for prospective RCTs. These should explore the above interventions and management techniques in high-risk infants with underlying medical comorbidities requiring ICU management.

## Conclusion

On review of current NICE guidelines for the management of acute bronchiolitis, we identified a paucity of evidence regarding the management of high-risk infants in the PICU, with the majority of RCTs excluding those with high-risk conditions, comorbidity and escalation of care. Our systematic review highlighted a similar lack of evidence-based management for high-risk infants with acute bronchiolitis in the PICU. Studies excluded patients on the basis of underlying medical disorders, prematurity, or did not account for potential adverse effects of interventions which may pose higher risk for vulnerable cohorts. In turn, those studies which did not exclude high-risk patients were conducted with smaller sample sizes. Although oxygen support is a mainstay of treatment in severe bronchiolitis, only one study identified did not exclude patients with underlying respiratory and cardiac conditions. We recommend further evidence-based trials into the use of prophylactic dexamethasone, heliox therapy, and early use of diuretic therapy, as these showed favourable outcomes. This review highlights the need for larger-scale, evidence-based trials on the above interventions, with recommended focus on prospective RCTs. Following on from this, future efforts can be tailored towards creating guidelines for the management of high-risk infants with acute bronchiolitis in the PICU setting.

## Supplementary information

Supplementary information

Supplementary

Supplementary
