# Supplementary material for: Bronchiolitis: evidence-based management in high-risk infants in the intensive care setting
Source: Pediatr Res. 2024 Jun 20;96(7):1560–7. doi: 10.1038/s41390-024-03340-y (PMC11772224; doi:10.1038/s41390-024-03340-y)
Supplement: Supplementary file 1 — Supplementary information [file 41390_2024_3340_MOESM1_ESM.pdf]

Table S1: Summary of the Categories of Interventions Identified in Bronchiolitis Management in Intensive Care.

Intervention	Number of included studies
**NPPV (CPAP or BiPAP)**	2
**HFNC**	5
**NPPV (CPAP or BiPAP) vs. Oxygen therapy (eg. HFNC)**	10
**Heliox**	2
**Dexamethasone**	1
**Nutritional supplementation**	1
**Ribavirin**	1
**RSV-IVIG**	1
**Diuretics**	1
**EPO**	1
**Caffeine**	1

Key: BiPAP: Bilevel Positive Airway Pressure; CPAP: Continuous Positive Airway Pressure; EPO: Erythropoietin; *HFNC: High-Flow Nasal Cannula Oxygen Therapy; NPPV: Non-Invasive Positive Pressure Ventilation; RSVIG: Respiratory Syncytial Virus Immune globulin/Immunoglobulin*

Table S2: Summary of the 26 Studies Extracted from our Search for Literature Regarding the Intensive Care Management of Bronchiolitis

See Excel File

Table S3: The Risk of Bias in each of the 26 Studies, Table 4a pertains to the 15 Trials which were assessed using the Cochrane risk-of-bias tool for randomized trials (Sterne et al. 2019). Table 4b pertains to the 11 Cohort Studies assessed using the Newcastle-Ottawa Scale (Wells et al. 2013).

Table S3a.

Study	Randomisation process	Deviations from the intended interventions	Missing Outcome Data	Measurement of the Outcome	Selection of the Reported Result
Jacobs et al. 2003	High risk	Some concerns	Low risk	Low risk	Some concerns
Milési et al. 2018	Low risk	Low risk	Low risk	Low risk	Low risk
Ergul et al. 2018	High risk	Low risk	Low risk	Low risk	Low risk
Mayordomo-Colunga et al. 2018	High risk	Low risk	Low risk	Low risk	Low risk
Sarkar et al. 2018	Low risk	Low risk	Low risk	Low risk	Low risk
Cesar et al. 2020	High risk	Low risk	Low risk	High risk	Low risk
Milési et al. 2013	High risk	Low risk	Low risk	Low risk	Low risk
Liet et al 2005	Low risk	Low risk	Low risk	Low risk	Low risk
Rodriguez et al. 1997	Low risk	Low risk	Low risk	Low risk	Low risk
Borgi et al. 2021	Low risk	Low risk	Low risk	Low risk	Low risk
Thia et al. 2007	Low risk	Low risk	Low risk	Low risk	Some concerns
De Betue et al. 2011	Low risk	Low risk	Low risk	Low risk	Low risk
Martinón-Torres et al. 2002	Some concerns	Low risk	Low risk	Low risk	Low risk
Guerguerian et al. 1998	Low risk	Low risk	Low risk	Low risk	Low risk
Pham et al., 2014	High risk	Low risk	Low risk	Low risk	Low risk

Table S3b.

Study	Selection of Study Groups	Comparability of Groups	Exposure/ Outcome	Overall Risk
Delacroix et al. 2020	4 stars	1 star	3 stars	8 stars
Habra et al. 2019	4 stars	0 stars	2 stars	6 stars
Kapur et al. 2019	3 stars	1 star	3 stars	7 stars
Heikkilä et al. 2018	4 stars	1 star	3 stars	8 stars
David et al. 2018	4 stars	2 stars	3 stars	9 stars
Dohna-Schwake et al. 2018	3 stars	1 star	3 stars	7 stars
Essouri et al. 2017	4 stars	1 star	3 stars	8 stars
Veldhoen et al. 2017	4 stars	1 star	3 stars	8 stars
Clayton et al. 2019	4 stars	2 stars	3 stars	9 stars
Metge et al. 2014	2 stars	1 star	2 stars	5 stars
Agasthya et al. 2021	2 stars	1 star	2 stars	5 stars

Figure S1: Search Strategy for Attainment of Studies Regarding Intensive Care Management of Bronchiolitis in Infants

(infant/exp OR 'infant' OR 'extremely premature infant/exp OR 'extremely premature infant' OR 'infant, extremely premature/exp OR 'infant, extremely premature' OR 'infant, premature/exp OR 'infant, premature' OR 'infant, premature, diseases/exp OR 'infant, premature, diseases' OR 'neonate, premature/exp OR 'neonate, premature' OR 'pre-mature infant/exp OR 'pre-mature infant' OR 'pre-term baby/exp OR 'pre-term baby' OR 'pre- term child/exp OR 'pre-term child' OR 'pre-term infant/exp OR 'pre-term infant' OR 'pre-term neonate'/exp OR 'pre-term neonate' OR 'pre-term newborn/exp OR 'pre-term newborn' OR 'premature/exp OR 'premature' OR 'premature baby'/exp OR 'premature baby' OR 'premature birth/exp OR 'premature birth' OR 'premature child'/exp OR 'premature child' OR 'premature childbirth/exp OR 'premature childbirth' OR 'premature infant/exp OR 'premature infant' OR 'premature infant disease/exp OR 'premature infant disease' OR 'premature infant diseases/exp OR 'premature infant diseases' OR 'premature neonate/exp OR 'premature neonate' OR 'premature newborn/exp OR 'premature newborn' OR 'premature syndrome/exp OR 'premature syndrome' OR 'prematuritas/exp OR 'prematuritas' OR 'prematurity/exp OR 'prematurity' OR 'preterm baby/exp OR 'preterm baby' OR 'preterm child'/exp OR 'preterm child' OR 'preterm infant/exp OR 'preterm infant' OR 'preterm neonate/exp OR 'preterm neonate' OR 'preterm newborn/exp OR 'preterm newborn' OR 'animals, newborn/exp OR 'animals, newborn' OR 'child, newborn'/exp OR 'child, newborn' OR 'full term infant/exp OR 'full term infant' OR 'human neonate/exp OR 'human neonate' OR 'human newborn/exp OR 'human newborn' OR 'infant, newborn/exp OR 'infant, newborn' OR 'neonate/exp OR 'neonate' OR 'neonatus'/exp OR 'neonatus' OR 'newborn/exp OR 'newborn' OR 'newborn animals/exp OR 'newborn animals' OR 'newborn baby'/exp OR 'newborn baby' OR 'newbom child/exp OR 'newborn child' OR 'newborn infant/exp OR 'newborn infant' OR 'newly born baby/exp OR 'newly born baby' OR 'newly born child/exp OR 'newly born child' OR 'newly born infant/exp OR 'newly born infant')
AND
(care, intensive/exp OR 'care, intensive' OR 'critical care/exp OR 'critical care' OR 'intensive care/exp OR 'intensive care' OR 'intensive care, paediatric/exp OR 'intensive care, paediatric' OR 'intensive care, pediatric/exp OR 'intensive care, pediatric' OR 'intensive therapy/exp OR 'intensive therapy' OR 'paediatric intensive care/exp OR 'paediatric intensive care' OR 'pediatric intensive care/exp OR 'pediatric intensive care' OR 'therapy, intensive/exp OR 'therapy, intensive')
AND
(bronchiolitis/exp OR 'bronchiolitis')
AND
('disease management/exp OR 'disease management' OR 'diseases management/exp OR 'diseases management OR 'disorder management/exp OR 'disorder management' OR 'disorders management/exp OR 'disorders management' OR 'illness management/exp OR 'illness management' OR 'management of disease/exp OR 'management of disease' OR 'management of disorder'/exp OR 'management of disorder' OR 'medical management/exp OR 'medical management')
AND
[english]/lim
AND
[clinical study]/lim
AND
([embase]/lim OR [medline]/lim OR [pubmed-not-medline]/lim)

Figure S2: Protocol for this Systematic Review

Research Question: What is the current evidence base for the management of bronchiolitis in infants admitted to intensive care?

Types of studies: Randomised controlled trials are the highest quality evidence and have the best internal validity, however due to the expected sparse nature of research in this field, we will also include cohort studies and case-control studies.

P (Population)	I (Intervention)	C (Control)	O (Outcome)
Infants <24 months admitted to a PICU for bronchiolitis treatment	A specified medical intervention for bronchiolitis (e.g. CPAP, HFNC etc.)	Infants not receiving the given intervention	superiority, inferiority, equivalence, non-inferiority

Types of participants: Infants under the age of 24 months with a diagnosis of bronchiolitis admitted to a paediatric intensive care unit (PICU). Infants could have underlying medical conditions and previous interventions.

Types of Interventions and Comparisons: Interventions - studies may have examined any intervention for bronchiolitis within the PICU, for example, the use of antibiotics, bronchodilators, hypertonic saline etc.

Comparisons - studies must also have controls which have a diagnosis of bronchiolitis and be admitted to a PICU who are not receiving the intervention being examined. The controls may be receiving alternative treatment.

Outcomes: The outcome measurements will vary across studies depending on the intervention used. We will include studies which report the outcome of the intervention used when compared to the control group in terms of superiority, equivalence, non-inferiority or inferiority.

Search Methods: Three databases will be used to conduct the literature search for this review: PubMed (National Institutes of Health), Medline (National Institutes of Health) and EMBASE (Elsevier). The following key words and any appropriate associated Emtree terms will be used in the literature search for this topic: Infant, Intensive Care, Bronchiolitis, and Disease Management. Only peer-reviewed literature will be preferentially chosen, provided a sufficient volume of papers can be obtained with this parameter. Only papers in the English language will be used in this study to ensure the accuracy of study selection and data extraction by preventing translation error. We will not enforce a timeframe in the search strategy as many older studies may still guide clinical management today.

Data Extraction and Analysis: Studies will be selected on the basis that they discuss the management of bronchiolitis in infants admitted to intensive care. Screening of studies will be divided between pairs of reviewers. The titles and abstracts of the studies will be screened using Covidence. Full-text screening will be conducted following the same process. A template for extraction of data will be created using Covidence, with the information regarding the intervention used, the exclusion criteria and the outcomes of the given paper. This data will then be compiled in Excel for ease of access for analysis. Data extraction will be carried out independently by the reviewers. Data analysis will be performed from this spreadsheet, to determine the proportion of studies that use various categories of interventions, the proportion of the studies which exclude similar underlying medical conditions as the RCTs within the NICE guideline reviews above, and the proportion of these studies which are RCTs or cohort studies. The evidence base produced will be summarised into a table of results for use by clinicians when examining the possible options for management of bronchiolitis in intensive care. This table will also highlight the gaps in the research which should be further explored.

Critical Appraisal: Assessment of Risk of Bias will be carried out using the Cochrane Risk of Bias Tool and the Newcastle-Ottawa Scale to assess bias across various domains within the studies. Overall bias across studies will be determined by combining assessments of each individual included study.
